# Editorial: Pharmacology of endocrine related GPCRs

**DOI:** 10.3389/fendo.2024.1379108

**Published:** 2024-02-15

**Authors:** Francesco De Pascali, Aylin Hanyaloglu, Frederic Jean-Alphonse, Francesco Potì, Eric Reiter

**Affiliations:** ^1^ Department of Biochemistry and Molecular Biology, Sidney Kimmel Medical College, Thomas Jefferson University, Philadelphia, PA, United States; ^2^ Institute of Reproductive and Developmental Biology, Department Metabolism, Digestion and Reproduction, Imperial College London, London, United Kingdom; ^3^ Physiologie de la Reproduction et des Comportements (PRC), Institut National de Recherche pour l’Agriculture, l’Alimentation, et l’Environnement (INRAE), Centre National de la Recherche Scientifique (CNRS), Université de Tours, Nouzilly, France; ^4^ Inria, Inria Saclay-Ile-de-France, Palaiseau, France; ^5^ Department of Medicine and Surgery, Unit of Neurosciences, University of Parma, Parma, Italy

**Keywords:** endocrine system, GPCRs (G protein-coupled receptors), gonadotropin receptors, pharmacoperones, ketogenic diet, prostaglandin E (PGE2) 2

About 30-40% of Food and Drug Administration (FDA)-approved drugs on the market target a G protein-coupled receptor (GPCR), qualifying these membrane-spanning proteins as an important pharmaceutical target (1). GPCRs are expressed in all tissues and control many different physiological functions by virtue of their ability to fine-tune cellular responses to endogenous and exogenous stimuli. GPCRs are activated when a stimulus (photons, odorant molecules, hormones, chemicals, etc.) interacts with the binding site (either orthosteric or allosteric), producing a characteristic conformational change in the GPCR structure that ultimately leads to an intracellular signaling cascade and a physiological output (2). At the same time, the endocrine apparatus is mainly structured as a glandular-hormone-tissue system in which a gland synthesizes the hormone that eventually modifies the function of a tissue. GPCRs are central in such system as they are the receiver of many of the circulating hormones (3). Consequently, issues with GPCR biology often result in endocrine diseases, which highlights the necessity to develop therapeutics to address GPCR-mediated endocrine disorders (4). In view of such unmet needs, this Research Topic aims to spotlight the most up-to-date advances in the pharmacology of endocrine-related GPCRs. It comprises four literature reviews encompassing basic molecular features of endocrine GPCRs and their clinical implications.

The review by Lazzaretti et al. focuses on the pharmacological modulation of gonadotropin receptors, a subfamily of the glycoprotein hormone receptor family that comprises the follicle-stimulating hormone receptor (FSHR) and the luteinizing hormone/choriogonadotropin hormone receptor (LHCGR). The two receptors and their hormones, follicle-stimulating hormone (FSH) for the FSHR, luteinizing hormone, and choriogonadotropin for the LHCGR, virtually control all aspects of human reproduction, both in males and females. Therefore, there is much interest in pharmacologically targeting these receptors for several applications, such as in assisted reproductive techniques, in contraception, or to cure/prevent reproductive-associated diseases. The authors describe a comprehensive set of molecules with different pharmacological properties that have been developed to selectively control gonadotropin receptor activity. These include agonists and antagonists, but the focus of the study is on the efforts made to develop suitable allosteric modulators to be used for clinical purposes. The authors describe all the different implications the allosteric modulation may produce on FSHR and LHCGR activity, as well as their influence on their cognate receptor’s structure. The authors conclude the review with some up-to-date insights into FSHR and LHCGR heterodimerization and how such structural occurrence can contribute to allosteric modulation.

In the review by Ulloa-Aguirre et al., the authors describe some specific endocrine disorders that originate from misfolded GPCRs that are unable to be located at the plasma membrane of the cells. Commonly, this happens when the cell quality control system (QCS), which is a complex protein system comprising chaperons, co-factors, enzymes, etc., identifies a GPCR that has an altered structure and reroutes its trafficking to degradation instead of the plasma membrane. Endocrine disorders associated with misfolded GPCRs are familial hypocalciuric hypercalcemia, X-linked nephrogenic diabetes insipidus, and familial glucocorticoid resistance, among many others. Furthermore, the authors center the discussion on the experimental strategies that have been implemented to restore the presence of the misfolded GPCRs partially or completely at the plasma membrane as an approach to treat the associated endocrine diseases. The authors concluded that one of the most promising and accessible strategies would be the one that employs pharmacoperones as treatment agents. These are molecules able to correct the folding of misfolded proteins, avoiding their accumulation or degradation by the QCS. They can be either agonists, antagonists, or allosteric modulators and might represent a winning strategy to cure endocrine diseases associated with missorted proteins.

The review by Spigoni et al. addresses the interplay between a ketogenic diet and the activation of endocrine-related GPCRs. The ketogenic diet is so-called because such an eating regimen leads to an overproduction of ketone bodies, which represent alternative energy sources for the human organism and a possible strategy for weight management. The authors highlight recent evidence supporting the activation of GPR109A, GPR41, and GPR43 by ketone bodies and their involvement in the endocrine control of lipid metabolism and inflammation. Indeed, the β-hydroxybutyrate (β-OHB) mediated activation of GPR109A provokes a decrease in adipocytic lipase activity, while the simultaneous activation of GPR43 by acetoacetate raises lipid consumption. Furthermore, the agonist activity of β-OHB on GPR109A may lead to anti-inflammatory effects, often associated with a ketogenic diet. In conclusion, with this review, the authors point out the pharmacological and molecular mechanism implications of GPCR activity with regard to some of the outcomes generally associated with a ketogenic regimen.

In the last review by Wang et al., the authors discuss the role of prostaglandin E2 (PGE2) in the endocrine regulation of fluid and blood dynamics. PGE2 is a lipid-derived hormone that controls a plethora of physiological functions. One of the most canonical is the inhibition of the activity of cyclooxygenases, resulting in the control of inflammation and pain. For the purpose of this review, the authors focus on the effects of PGE2 as an agonist of the four subtypes of the E-prostanoid (EP_1-4_) receptors in the control of hemo-and fluid dynamics in the various cell and tissue types where the EP receptor subtypes are differently expressed. Through a comprehensive analysis of the available scientific literature, the authors conclude that PGE2 mediates fluid and blood pressure homeostasis in either a positive or negative manner. Furthermore, the four EP receptor subtypes are responsible for selectively contributing to the different outputs, putting them at a glance to be possible targets of endocrine disorders related to fluid and blood dynamics.

In summary, the four review articles that constitute this Research Topic highlight the important role GPCRs play in regulating the endocrine system. The pharmacological targeting of such receptors is an attractive approach that researchers and clinicians are interested in pursuing. At the same time, more effort is needed to understand the basic biology and pharmacology of GPCRs as a starting point to develop safer and more efficacious drugs for the treatment of endocrine disorders.

## Author contributions

FP: Writing – original draft, Writing – review & editing. AH: Writing – original draft, Writing – review & editing. FJ-A: Writing – original draft, Writing – review & editing. FP: Writing – original draft, Writing – review & editing. ER: Writing – original draft, Writing – review & editing.
